# Pandemics and food systems - towards a proactive food safety approach to disease prevention & management

**DOI:** 10.1007/s12571-020-01074-3

**Published:** 2020-07-10

**Authors:** Anaka Aiyar, Prabhu Pingali

**Affiliations:** 1 Department of Economics, University of Nevada, Reno, 1664 N. Virginia Street, Reno, NV 89557 USA; 2 Tata-Cornell Institute for Agriculture and Nutrition, Dyson School of Applied Economics & Management, Cornell University, Ithaca, NY 14853 USA

**Keywords:** Covid19, Food safety, Food systems, Biosecurity, Policy

## Abstract

Recent large-scale pandemics such as the covid19, H1N1, Swine flu, Ebola and the Nipah virus, which impacted human health and livelihoods, have come about due to inadequate food systems safeguards to detect, trace and eliminate threats arising from zoonotic diseases. Such diseases are transmitted to humans through their interaction with animals in the food value chain including through the consumption of bush meat. Climate change has also facilitated the emergence of new zoonotic diseases. The lack of adequately enforced food-safety standards in managed agricultural production systems creates the necessary conditions for diseases to mutate into highly contagious strains. The lack of food safety measures in handling, packaging and sales of food increases risks of cross-species contamination. Finally, increasing anti-microbial resistance, combined with rapid urbanization and global interconnectedness allows diseases to spread rapidly among humans. Thus, part of the reconstruction efforts, post covid19, should include prioritizing proactive investments in food safety. The key to stave off another such pandemic lies in integrating one-health knowledge on zoonotic diseases along with food safety measures along the food value chain. Refocusing policy priorities from disease control to prevention will improve international coordination efforts in pandemic prevention. Implementing such proactive actions will cost a very small fraction of the reconstruction budgets. However, the expected benefits of the food-safety approach will include preventing global economic losses due to pandemics.

## Introduction: The food systems origins of pandemics

**Fig. 1:**
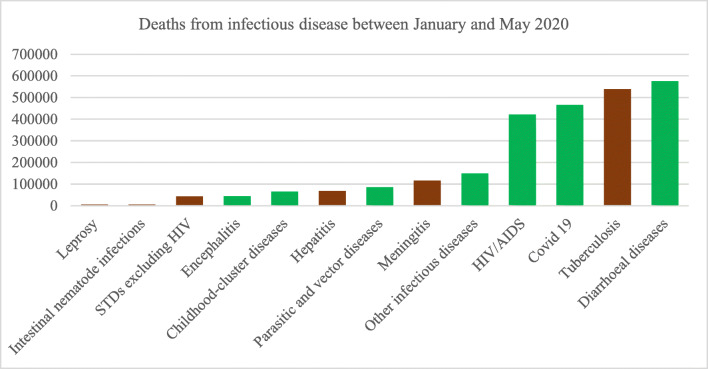
As of May 31st^,^ 2020, Covid19 was among the top 3 zoonotic disease killers across the globe

According to the Global Health Estimates produced by the World Health Organization (WHO), 20% of all global deaths could be attributed to infectious communicable diseases. Of these, 50% of the top 5 killers were zoonotic in origin (Fig. 1, green bar).^1^ The last two decades has seen a rise in the number of pandemics that have had their origins in zoonotic diseases. The PREDICT project at USAID has documented at least 900 new strains of pandemic causing infectious diseases in this time.^2^ Some of these diseases that have escalated into pandemics such as the SARS (2003), Nipah (2009), Ebola (2014) and now the Covid19, have emerged from animal-human interactions in the food-system (Gebreyes et al. 2014; Kelly et al. 2017; Lu et al. 2020; Marty and Jones 2020). These pandemics have caused losses to economic wellbeing and food security across the world (Smith et al. 2019).

Zoonotic diseases, transmitted to humans from animal hosts, have emerged due to the lack of monitoring and enforcement of food-safety standards in the food systems. Improper storage of animals, unsanitary conditions and poor handling of livestock products in managed production systems and wildlife trade have been identified as the channels for mutation of viral strains and for cross-species transmissions (Cutler et al. 2010; Destoumieux-Garzón et al. 2018; Han et al. 2016; Hassell et al. 2017; Kruse et al. 2004; Parrish et al. 2008). Supported by human to human transmission due to urbanization, growing ease of local and global transport and growing anti-microbial resistance, viruses such as novel coronavirus have found opportunity to escalate into pandemics (Holmes et al. 2016; Morse et al. 2012).

Much of the global response to zoonotic disease emergence continues to be reactionary. The political community is currently focused on containment and economic reconstruction. The food security community is focused on ensuring continued food access. Human-health experts are focused on treating the disease and identifying a vaccine. Economic experts focus on alleviating negative impacts associated with loss of GDP and livelihoods. While these responses are vital in this post-covid era, we urge that designing, promoting and enforcing food-safety measures is an *urgent and proactive* measure to protect against such unintentional biosecurity threats. One-health experts have long recognized that active and engaged zoonotic disease surveillance is crucial to stop infectious disease outbreaks (Destoumieux-Garzón et al. 2018; Kelly et al. 2017; Zinsstag et al. 2011).^3^ In this paper we highlight evidence that a proactive-response strategy is imperative to prevent pandemics like Covid19. Taking proactive steps to incorporating one-health expertise along with food-safety interventions will reduce the risks of emergence of new diseases. Global interest in containment of this disease should be directed towards changing behaviors and policies in favor of proactive efforts. Enhancing local government capacity will be essential to ensure that programs are properly implemented and monitored. These inputs can simultaneously reduce threats from infectious diseases and bolster food and economic security.

### Zoonotic disease risks in managed production systems

**Fig. 2:**
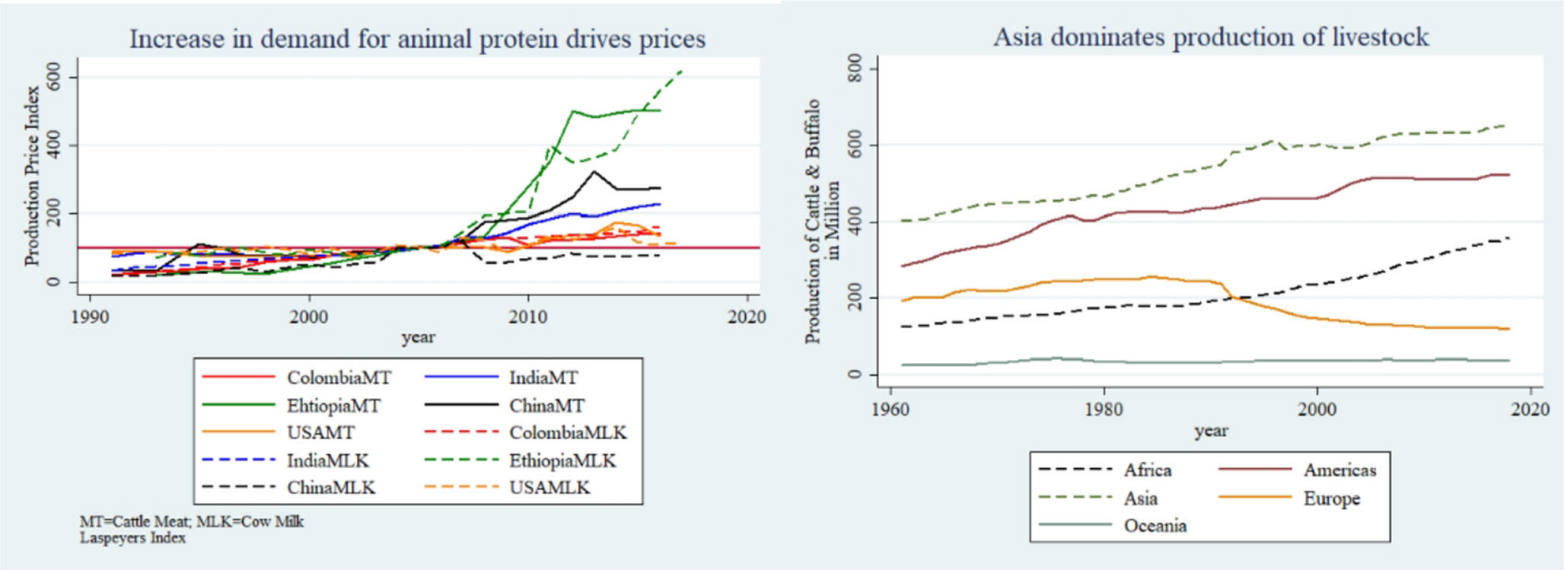
Panel A - We see the demand effect of westernization of diets. Prices of meat and milk have been increasing in the developing world but have remained somewhat constant in developed countries (orange lines). Panel B - we show that majority of the livestock produce comes from developing countries with Asian producers dominating production and exports

Westernization of diets and increased demand for dietary diversity in many developing countries (Pingali 2007) has driven up prices of livestock products (Fig. 2 Panel A).^4^ Facilitated by economies of scale in agricultural production, livestock production has intensified (Chavas 2008; Duffy 2009). In Asia, land constraints for production have been overcome with technological innovations to boost livestock productivity (Steinfeld et al. 2006). Currently, more than half of the world’s livestock is produced in Asia with Chinese producers dominating most of the global markets (Fig. 2, Panel B).^5^

Diseases - such as the swine flu, avian flu and hoof and mouth disease which affect pigs, chicken and cows respectively - have emerged due to the lack of adequate sanitation and hygiene practices in the livestock industry (Leibler et al. 2017; Steinfeld et al. 2006). For example, the highly contagious strain of avian flu virus has been found in overcrowded chicken farms (Gauthier-Clerc et al. 2007).^6^ Containing the pandemic cost Vietnam and Thailand around USD 45 million and USD 135 million. The disease destroyed food supplies. Nearly 18% of Vietnam’s total poultry livestock and 15% of Thailand’s livestock was culled to contain the outbreak in 2004 (Burgos and Burgos 2007). Exposures and deaths among humans were traced to unsafe hygiene practices with disposing infected poultry and carcasses (Gauthier-Clerc et al. 2007).^7^

Productivity enhancements to maintain competitiveness in managed-livestock production systems have reduced genetic diversity in livestock breeds. Overuse of antibiotics in production to stimulate growth and protect herd health has been found to increase anti-microbial resistance among humans. The channels include direct consumption of livestock products or indirect contact during livestock processing (Landers et al. 2012; Levy et al. 1976; Marshall and Levy 2011; Price et al. 2007). The rise in anti-microbial resistance poses significant challenges for fighting new bacterial threats that will emerge.

Lack of proper sanitation and hygiene while handling animals and their products has been identified as a mechanism for cross-species transmission. The emergence of the Nipah virus in Malaysia has been linked with the contamination of swine feed with saliva and feces of bats (Chua 2003). Direct exposure through improperly stored date-palm water and indirect contamination through exposure to sick livestock enabled transmission to humans in Bangladesh (Hughes et al. 2009; Luby et al. 2006). Costs to contain Nipah in Malaysia included USD 97 million from culling affected swine populations, USD 136 million in health system responses, and around USD 229 million in tax revenues due to losses in industries such as tourism (Smith et al. 2019).

### Zoonotic disease risks arising from human-animal interactions in wet markets

Improper storage of animals, overcrowding, inadequate hygiene and improper disposal of feces, and improper disposal of carcasses in wet markets has been linked to cross-species transmission of infectious diseases. The lack of enforcement of food-safety standards has allowed such wet markets, that trade livestock products locally, to become infectious disease hot spots.

In subsistence-based production systems livestock trade supplements incomes and provides meat and nutrition for the poor. Markets for meat are mostly unregulated and are locally oriented. Direct contact with infected wildlife carcasses, without safety equipment, led to emergence of Ebola (Leroy et al. 2004). To date, nearly 30,000 individuals have been infected with a case death-rate of around 50%.^8^ The estimated cost of containing the pandemic was around USD 1 billion by the end of 2014 (Butler and Morello 2014). GDP growth in Sierra Leone, Liberia and Guinea fell to less than 1% after the outbreak (Smith et al. 2019).

The SARS-Cov and novel coronavirus have their origins in illegal wildlife trade. The SARS-Cov, with a case death rate of 10%, emerged from viral transmission of a strain of coronavirus from bats to humans, through an intermediary host, civet cats (Li et al. 2006; Lu et al. 2020; Shi and Hu 2008). The SARS-Cov pandemic is thought to have cost Asia-pacific air carriers USD 6 billion. Hong Kong’s tourism industry reduced by more than 60% and its GDP fell by 2.6% in 2003. The estimated costs of containment of the SARS ranges between USD 10–50 billion (Smith et al. 2019). The novel coronavirus, the architect of the Covid19 pandemic, has been linked to wildlife trade in Wuhan. Lack of adequate sanitation and improper storage of animals alongside wildlife is thought have created the conditions for its transmission to humans.^9^ In addition to 11.8 million individuals who have been affected this far, and with a case-death rate of 5%, controlling the spread of the virus and restarting the economy is expected to cost the world at least 1% of its GDP in 2020.^10^ This calculation does not include the indirect losses from reduction in trade and aggregate demand.

### Other risks for zoonotic disease – Impacts of biodiversity loss and climate change

New threats of such diseases emerging have been augmented by biodiversity loss and climate change. Deforestation, for example, forces humans to come in contact with new types of animals that carry disease vectors that are otherwise harmful for human health (Jones et al. 2013; Morse et al. 2012; Parrish et al. 2008). Long term climate change also poses challenges for the re-emergence of previously eliminated communicable diseases from changing ecological environments (Gould and Higgs 2009). The virulence of Eschiera Coli, a bacteria that causes gastroenteritis, is positively correlated with temperature and precipitation. The e-coli pandemic has been transmitted via the food value chain across the developed world and through poor sanitation and hygiene practices in the developing world (Liu et al. 2013).

## Food systems characteristics that enable outbreaks snowballing into pandemics

Covid19 has brought to our attention that local outbreaks can morph into global pandemics within a short period of time. More than half the world is now living in urban areas (Pingali and Aiyar 2018) with high population density (Hassell et al. 2017). This, along with poor sanitation and hygiene practices, has enabled faster transmission of disease from animal hosts to humans and between individuals (Clasen et al. 2014). Ease of travel, by land and air, has facilitated the rapid transmission of diseases within a country and across countries.

### The food-safety blind spot of food systems

There has been interest in enforcing internationally compatible food safety standards to ensure continued global food trade (Brown et al. 2002; Kirezieva et al. 2015; Nguyen-Viet et al. 2017). At the local level, private sellers determine compliance with standards (Hammoudi et al. 2009; Henson and Humphrey 2010; Henson and Reardon 2005). Richer consumers who can pay higher prices for quality purchase produce from formal retailers. These retailers are more likely to enforce government-regulated safety standards. Consumers who buy from the wet markets tend to be poor and rely on their social relationships with sellers to ensure food-safety standards are enforced (Grace et al. 2014; Roesel and Grace 2014). This creates variability in how standards are enforced in local produce markets. Additionally, demand for illegally poached wildlife pose problems for enforcement of food-safety. In the absence of a legitimate market, enforcement of food safety standards in storage and handling of animals is not possible. This oversight creates a convenient blind spot in local food systems where viruses, such as the coronavirus, can thrive.

## A food safety approach for pre-emptive action against future pandemics

The emergence of Covid19 re-emphasizes that enforcing food-safety standards in local food systems should be a global priority. Our experience shows us that poorly enforced standards have cross-industry and cross-country ramifications. Pre-emptive action requires a recognition that food-safety is a *global* public good. In the absence of external interest in implementing food-safety as a precautionary measure, countries may continue to underinvest in this aspect of food systems (Henson and Traill 1993; Unnevehr 2007).

### Market based approaches for aligning one-health goals with food-safety directives

Increasing demand for food-safety as a product attribute will increase adoption of such practices at the farm and in markets (Pouliot and Sumner 2008; Wang et al. 2008). Willingness to pay for on-farm diversification, lower antibiotic-use and sanitary handling of food will incentivize local producers to adopt such food-safety directives. Demand push can be facilitated by education and through the enforcement of reliable and consistent food-safety measures which are monitored by the government (Caswell and Mojduszka 1996; Loureiro and Umberger 2007). This will not only reinforce consumer willingness to pay for food-safety, it will ensure greater traceability of disease in the value chain. Enforcing penalties for violation of food-safety measures by large food processing firms could speed up adoption of such practices. Extension and training on food safety standard and practices targeted towards small firms can help them become competitive relative to larger firms.

Wet markets play an important role in enhancing incomes and livelihoods of poor smallholder farmers. It is important to ensure that the introduction of new food safety standards does not disrupt their access to wet markets. Governments can play a proactive role by investing in clean water and sanitation infrastructure for wet markets. Training market suppliers and sellers in basic food safety and food handling practices and frequent monitoring of their use would also be a crucial role for local government agencies. Early identification of disease outbreaks requires cooperation of livestock owners and sellers. Data from monitoring of animal health needs to be integrated with epidemiological studies on health risks. Providing financial instruments such as insurance against zoonotic diseases will incentivize food-safety adoption for risk management (Meuwissen et al. 2006). Understanding the socio-economic factors that characterize current infectious hotspots can help identify effective market instruments for adoption of disease surveillance efforts both on the farm and in the wet markets.

### Sanitation and hygiene for greater food safety and one-health

Enforcing sanitation and hygiene standards at the farm and in markets is an important step for greater food-safety as well as for the reduction of infectious disease. This involves incentivizing investment in water and sanitation (WASH) infrastructure at production houses and markets. Ensuring that animals and meat are stored appropriately requires investment strategies to bolster storage infrastructure and cold chains (Grace et al. 2014; Rosegrant and Cline 2003; Steinfeld et al. 2006). Behavioral changes among producers should be a focus of food-safety interventions as well. Information and education programs should encourage better hygiene practices. Identifying if the key constraint is infrastructure, resource or knowledge would be important for ensuring such hygiene improving interventions are cost-effective. Incorporating food-safety information into behavior change communication programs around WASH behavior for households can stimulate local demand for food-safety practices and facilitate disease prevention efforts.

### Integrating traceability technologies at local disease hotspots

From our experience of recent pandemics, a major goal for containment is reducing the time delays in identification of diseases at its source. Thus, some funds allocated for reconstruction efforts should be directed towards the development of technology to track, trace and eliminate zoonotic diseases. Real time health data, collected through smart phones and watches, can be leveraged to identify emerging outbreaks in both humans and animals. Utilizing internet search histories based on frequency of health queries in disease hotspots can be utilized as well. Historical data on zoonotic diseases along with socio-economic information on livestock production and consumption patterns at the disaggregated level can be integrated into data analytics software. These databases can be used for health monitoring and disease surveillance. Local food labs that test food quality should be equipped with surveillance technology and given access to data repositories to identify threats coming from food value chains. One-health researchers and food-safety experts need to work alongside epidemiologists and local food-systems stakeholders to bolster surveillance efforts.

### De-politicizing zoonotic disease emergence by focusing on local food safety as a key reconstruction goal

At the country level, integrating food-safety goals for disease prevention along with food safety directives in trade will help enhance trade and maintain competitiveness while simultaneously incentivizing locally driven containment efforts. The Codex Alimentarius, an international food safety standard protocol is one such directive that can be used to help countries meet the twin goals of equitable trade and better health through food-safety investments.^11^ To be successful in containment, local stakeholders need to be actively engaged in both implementation of the standards as well as in active surveillance (Häsler et al. 2013). Disease spread can be controlled only if there is transparency in sharing information and if local actors are empowered with resources to act on disease threats. Strengthening inter-departmental coordination between the one-health, health and food systems groups can reduce time lags in disease identification and in implementing containment policies (Kelly et al. 2017; Morse et al. 2012). In areas where research capacity is the major constraint, global actors can play a role in supplementing the local knowledge base.

### Conservation as a long-term food safety measure

Food-safety experts should encourage research in maintaining genetic diversity of livestock. Investments in vaccine development and development of veterinary services for livestock are also important inputs into animal health. These interventions can reduce livestock susceptibility to zoonotic diseases and reduce threats of rising anti-microbial resistance (Holmes et al. 2016; Marshall and Levy 2011). Increasing efforts towards conservation either through demand reduction of wildlife and/or promotion of afforestation will reduce exposure to species that carry dangerous disease vectors (Hassell et al. 2017). Such efforts will be important to reduce transmission of high-risk but low-probability zoonotic diseases.

## Discussion & Conclusion

A major take away from the emergence and spread of the Covid19 is that food systems have become more vulnerable to disruptions due to the emergence of infectious zoonotic diseases. Managing human-animal interactions at their source is at the heart of containment of such disruptions. On the production side, improving livestock health requires attention from one-health experts and more research in veterinary services. Vaccinations of livestock and reduction in the use of antibiotics will be effective in both improving animal as well as improving human health. In order to overcome the problem that social benefits & costs of food-safety outweigh private benefits and costs of implementing such practices, economic incentives need to be bolstered by efforts to facilitate behavior change. Encouraging WASH practices through social and behavioral interventions and social mobilization will be important to incentivize farmers to internalize social costs of underinvesting in food-safety practices on the farm.

Proper handling of food, improving storage quality for livestock and food products and enhanced packaging standards will reduce cross-species transmission. The food value chains in developing countries need to be carefully monitored. Introducing food-safety labels and educating consumers on the importance of safe food will increase demand and hence willingness to pay for food safety as a product attribute. This will speed up adoption of food safety measures in the supply chain.

Between SARS-Cov, Ebola and Covid19, the threats to human life and economic losses have increased multi fold. Increase in speed of local and global trade is the main catalyst in this risk. Thus, going forward, integrating traceability technology in monitoring disease emergence especially in places where animals and humans interact is a major requirement. This will help overcome the time risks that global trade presents to pandemic spread. Technology can be used to collate and distribute information quickly, both locally and globally, and can be effective in tracking when used appropriately. However, for technology to be effective there needs to be greater data sharing between local and global research units that are focused on studying such issues.

Investing in the development of local government capacity to track, trace, contain and prevent such diseases will help reduce global transmission. Pandemic control also requires coordination between the farming community, epidemiologists, animal science researchers, wet market traders, exporters, local businesses and consumers. To prevent underinvestment at the national levels, such efforts must be coordinated by global actors. However, it is also safe to assume that without simultaneous investments in bio-diversity conservation, many of these initiatives may fall short in the long term. Hence a glocal-long term approach to food safety is imperative in preventing future pandemics and food systems disruptions. Food-safety investments today will be extremely cost-effective reconstruction-related investments.

## Data Availability

Appropriate data sources have been cited. Authors agree to make this available on publication of the piece.

## Footnotes

1 Based on authors calculations from the Global Health Estimates 2016. A database collated by the world health organization. The database was downloaded in April 2020. Covid19 deaths estimates reflect deaths as of 30th April 2020. To make the data on deaths comparable between covid19 and annual figures in the GHE, we have presented approximate numbers of deaths that took place in 4 months in the year.

2 https://ohi.vetmed.ucdavis.edu/programs-projects/predict-project

3 “One Health is a collaborative, multisectoral, and transdisciplinary approach — working at the local, regional, national, and global levels — with the goal of achieving optimal health outcomes recognizing the interconnection between people, animals, plants, and their shared environment.” – Center for Disease Control & Prevention, https://www.cdc.gov/onehealth/basics/index.html, Accessed June 2020

4 http://www.fao.org/faostat/en/#data/PI - Producer Price Indices collated from the food and agricultural organization in April 2020.

5 http://www.fao.org/faostat/en/#data/QL - Livestock Primary indicators collated from the food and agricultural organization in April 2020.

6 Low pathogenic strains of the virus can be found in wild birds and trace their pattern of migration. The high pathogenic strains are commonly found in domesticated birds and the disease can be traced using information on domestic and international trade patterns.

7 The main reason the human impact of this disease was not as large as Covid19 was that the virus’s spread was limited by a lower R0. However, even today, the WHO continues to track this disease and regularly issues warning on local outbreaks. https://www.who.int/news-room/fact-sheets/detail/influenza-(avian-and-other-zoonotic)

8 https://www.who.int/news-room/fact-sheets/detail/ebola-virus-disease

9 At the time of writing this article, the WHO has been maintaining its stand that the disease has zoonotic origins.

10 Accessed 8th July 2020 - https://coronavirus.jhu.edu/map.html

11 The Codex Alimentarius was developed jointly by the FAO and the WHO. http://www.fao.org/fao-who-codexalimentarius/en/
